# Evolutionary Origins of Cancer Driver Genes and Implications for Cancer Prognosis

**DOI:** 10.3390/genes8070182

**Published:** 2017-07-14

**Authors:** Xin-Yi Chu, Ling-Han Jiang, Xiong-Hui Zhou, Ze-Jia Cui, Hong-Yu Zhang

**Affiliations:** Hubei Key Laboratory of Agricultural Bioinformatics, College of Informatics, Huazhong Agricultural University, Wuhan 430070, China; chuxy@webmail.hzau.edu.cn (X.-Y.C.); jianglinghan@hotmail.com (L.-H.J.); 18202782627@163.com (Z.-J.C.); zhy630@mail.hzau.edu.cn (H.-Y.Z.)

**Keywords:** cancer, driver genes, evolution, endogenous molecular network, prognosis

## Abstract

The cancer atavistic theory suggests that carcinogenesis is a reverse evolution process. It is thus of great interest to explore the evolutionary origins of cancer driver genes and the relevant mechanisms underlying the carcinogenesis. Moreover, the evolutionary features of cancer driver genes could be helpful in selecting cancer biomarkers from high-throughput data. In this study, through analyzing the cancer endogenous molecular networks, we revealed that the subnetwork originating from eukaryota could control the unlimited proliferation of cancer cells, and the subnetwork originating from eumetazoa could recapitulate the other hallmarks of cancer. In addition, investigations based on multiple datasets revealed that cancer driver genes were enriched in genes originating from eukaryota, opisthokonta, and eumetazoa. These results have important implications for enhancing the robustness of cancer prognosis models through selecting the gene signatures by the gene age information.

## 1. Introduction

The genesis and progression of cancer are evolutionary processes [1,2,3]. Based on the hallmarks of cancer cells, such as the breakdown of cooperation between cells and the unlimited replication potential, the atavistic theory of cancer was established, which proposed that in these processes, cancer cells use the ancient toolkit that services unicellular survival [4,5]. This theory also suggested a link between cancer and the early stage of macroevolution. For instance, it was reported that the cancer-related genes were enriched in the evolutionary transition from unicellular to multicellular organisms, and the multicellular-related genes could promote cancer metastasis in a loss-of-function way [6,7]. Bezginov et al. found that cancer genes were enriched in a protein–protein interaction network representing functions evolved in multicellular animals [8]. These studies provide evidences for the link of cancer and the origin of multicellular organisms. However, the gene age estimation method used in prior studies has inherent bias, which may impair the downstream analyses [9,10].

Recently, Liebeskind et al. provided a consensus gene age data [10]. The gene age in this data set was speculated based on thirteen popular orthologue inference algorithms, thus reducing the bias of a single algorithm. In this study, this data set was used to trace the origin of cancer driver genes. On the other hand, genes involved in carcinogenesis do not work separately but function in networks. So, a systematic description of the functions of the networks constructed by genes originating from certain evolutionary stages will deepen the understanding of the link of cancer and macroevolution.

The cancer endogenous molecular network theory proposed by Ao’s group provided a tool to investigate the mechanisms underlying carcinogenesis on a systematic level [11,12,13]. The network was constructed by molecular factors that related to cancer hallmarks and reflected the main characteristics of cancer. The regulatory relationships among these factors were represented by a series of nonlinear differential equations. The descriptions of the mechanisms of carcinogenesis were represented by the attractors generated from the stochastic nonlinear dynamical systems. The accuracy of the descriptions could be verified by the real biological data. This method has been tested in different cancers and has provided accurate descriptions of mechanisms of carcinogenesis [14,15,16,17]. The above-mentioned progresses provide us with an opportunity to trace the evolutionary origins of cancer driver genes and investigate how they promote carcinogenesis from a systematic viewpoint.

In recent years, high-throughput data are widely applied in cancer research. Cancer biomarkers derived from these data were usually selected by the statistical association. However, in these processes, the biological relevance between the biomarkers and cancer is often overlooked, this may lead to the generation of false-positive biomarkers [18]. Liu et al. used genes’ evolutionary conservation information as prior knowledge in cancer biomarker selection, and showed that the evolution-informed biomarkers performed better than the statistical biomarkers in predicting the therapeutic responses in acute myeloid leukemia and the metastasis in prostate cancer [19]. This study suggests that evolutional information may have implications for improving the accuracy of cancer biomarker selection.

In this work, we constructed cancer endogenous subnetworks originating from different evolutionary stages and investigated their functions. Then, we examined the enrichment pattern of cancer driver genes in different evolutionary stages. Finally, we integrated the evolutionary features of cancer driver genes into cancer biomarker selection and evaluated the performances of the biomarkers in three cancer prognosis models.

## 2. Materials and Methods

### 2.1. Endogenous Network Analysis

#### 2.1.1. Construction of the Endogenous Networks

We constructed the subnetworks based on the endogenous networks of hepatocellular carcinoma [14], gastric cancer [15] and prostate cancer [16]. In the above studies [14,15,16], the construction of cancer endogenous networks was started with function modules that were related to cancer hallmarks such as cell cycle, glycolysis, apoptosis, cell adhesion, immune response and angiogenesis. Then, the genes which played a key role in each function module were selected as candidate nodes. The information of gene functions and regulation relationships among these genes were obtained by searching relevant literatures in PubMed database (https://www.ncbi.nlm.nih.gov/pubmed/). Based on this information, the networks were manually constructed. Next, the networks were quantified (described in Section 2.1.2), and the accuracy of the results was evaluated after compared with experimental data (described in Section 2.1.3). If the accuracy of the simulation result cannot achieve an acceptable level (normally over 70%), nodes were removed or added to the networks. Then the above process was repeated, until the accuracy of the result achieves an acceptable level. The final networks were considered to be able to reflect the core regulation mechanisms underlying carcinogenesis.

In this study, nodes which existed in at least two of the final networks of the three studies mentioned above were selected as candidate nodes. This step can remove some tissue-specific genes to make sure that the networks we constructed could represent the common features of cancer. Then, the candidate nodes were mapped to the consensus gene age data. For the nodes which were composed by multiple proteins, their ages were considered to be the same as the youngest protein. We chose the nodes that were originating from eukaryota, opisthokonta and eumetazoa from the candidate nodes to build the multicellular subnetwork (Table S1). Then, we chose the nodes originating in eukaryota to build the eukaryotic subnetwork (Table S1). We added Integrin/FAK complex into the eukaryotic subnetwork because the homologs of these two proteins have been identified in yeast [20,21]. The regulation relationships among these nodes were set the same as the original works did [14,15,16], which means the structure of the original networks was kept. The regulatory relationships among these nodes are listed in Table S1.

#### 2.1.2. Quantification of the Endogenous Networks

The network quantification method we used was the same with the method used in previous studies [14,15,16]. The regulatory relationships between nodes are from the three networks mentioned above (Table S1). The regulation relationship is described by Equation (1):

(1) $$\frac{dxk}{dt}=f(x_{i},\;x_{j})-\frac{xk}{\tau xk}$$

where *x_k_* is a certain node, and *x_i_* and *x_j_* are nodes regulating *x_k_*. *x_k_* represents the activity/concentration of *x_k_*, $\frac{xk}{\tau xk}$ represents the degradation rate of *x_k_*, and *τ_xk_* is the degradation constant of *x_k_*. It is very difficult to measure the degradation constant of all of the proteins involved in the network, so the parameter was simplified to 1 in cancer endogenous network analysis [14,15,16]. *f*(*x_i_*, *x_j_*) represents the integrated production rate of *x_k_* that is modeled by the sigmoid-shaped Hill functions. We suppose that *x_k_* is activated by *x_i_* and inhibited by *x_j_*, and the Hill functions to describe activating and inhibiting regulations are Equation (2):

(2) $$factivation=\frac{a{\left[x_{i}\right]}^{n}}{1+a{\left[x_{i}\right]}^{n}},\;finhibition=\frac{1}{1+a{\left[x_{j}\right]}^{n}}$$

where *n* represents the Hill coefficient, and *a* represents the kinetic properties of each component *x*. Previous studies have found that the switch-like input/output relationships are dominant in cell signaling [22] and the cancer endogenous network theory assumed that interactions among the nodes of the network should show a switch-like behavior [14,15,16]. Li et al.’s work proved that the system showed a switch-like behavior only when 3 ≤ *n* ≤ 10; when *n* is in this range, the attractors generated under different *n* values are substantially equivalent [15]. As for the other parameter *a*, the work also proved that *a* ≈ 2*^n^* [15]. We adopted *n* = 3 and *a* = 8 in this study. Other combinations of this parameter have also been tested; however we found that the results of these simulations are equal to the result when *n* = 3 and *a* = 8 (data not shown). Here we take BAX as an example to illustrate how to construct a differential equation for a node. In the endogenous network we constructed, BAX is activated by c-Myc and p53 while it is inhibited by Bcl-2 and Bcl-xL. Using the above two equations we can form the Equation (3):

(3) $$\frac{{dx}_{BAX}}{dt}=\frac{a*x^{n}{}_{c-Myc}+a*x^{n}{}_{p^{53}}}{1+a*x^{n}{}_{c-Myc}+a*x^{n}{}_{p^{53}}}*\frac{1}{1+a*x^{n}{}_{Bcl-2}+a*x^{n}{}_{Bcl-xL}}-x{}_{BAX}$$

After forming the equations for all of the nodes, the attractors of their network were calculated using the fixed-point iteration algorithm used in Li’s study [15]. The nonlinear dynamical system is described by Equation (4):

(4) $$\frac{dv(t)}{dt}=f(v)$$

here, ***v*** is the vectorized *x*, which consists of relative activities/concentrations for all the nodes in the network. The activities/concentrations were normalized within 0 to 1, where 0 means minimal activities/concentrations and 1 means maximal activities/concentrations. We start with a vector ***v*_0_** which consists of random activities/concentrations of the nodes in the network. *v* was iterated in the dynamical systems using Equation (5):

(5) $$v_{l+1}=v_{l}+\Delta t\times f(v_{l})$$

After 40,000 times of iteration, the convergence was judged by $\left|v_{l+1}-v_{l}\right|<\varepsilon$ and $\left|f(v_{l})\right|<\delta$, here *δ* = 0.0001 and we recorded the convergent ***v*** that meets the definition as an attractor.

#### 2.1.3. Accuracy Evaluation of the Endogenous Networks

By the above step, the attractors, which were convergent vectors, could characterize the relative activities/concentrations of the nodes. In previous studies [14,15,16], according to the states of function modules and pattern of the nodes’ activities/concentrations in an attractor, the corresponding cell status of the attractor could be determined [14,15,16]. For example, if the genes which activate cell proliferation showed high activities/concentrations while the genes which inhibit cell proliferation showed low activities/concentrations, the cell cycle module was regarded as being activated and vice versa. Other functional modules’ states were also evaluated in this way. For an attractor, if the modules related to cell cycle, glycolysis, immune response and angiogenesis were active while the modules related to apoptosis and cell adhesion were inactive, it was determined as a cancer-like cell status. In this way, the attractors could be classified into four types, including proliferation (cancer-like), cell cycle arrest (be regarded as the normal-like status. In this status, cell adhesion is active and the other modules are inactive), apoptosis (apoptosis is active and the other modules are inactive), and stress response (immune response is active). The attractors and corresponding cell status information, which were derived from the previous studies [14,15,16], are summarized in Table S2. If the attractors were not consistent with any cell status, the network should be modified as we described above.

After the cell status of each attractor was determined, the change of the relative activities/concentrations of nodes between attractors could be compared with corresponding experimental data. That is, the difference of the nodes’ activities/concentrations between the cancer-like attractor and the normal-like attractor was compared with the difference of the corresponding genes’ expression levels between the cancer tissue and the normal tissue. In the previous studies [14,15], gene expression data were obtained from multiple microarray expression profiling data of cancer and its adjacent noncancer tissues. According to the average difference of a gene’s expression level between cancer and normal tissue, the node that was encoded by the gene was annotated as “Up-”, “Down-” or “Unchanged-” expressed in cancer. This annotation information was also used in this study. The percentages of nodes that showed consistent changes between the simulation results and the experimental results were calculated, which were used to reflect the simulation accuracy. If the accuracy does not reach an acceptable level, the network should be modified as we described above.

In this study, we determined the corresponding cell status of attractors generated from our subnetworks by comparing their nodes’ activities/concentrations pattern with the cell status of the attractors generated from the endogenous networks [14,15,16] (Table S2). Then, the change of nodes’ activities/concentrations between cancer-like and normal-like attractors were compared with the annotated expression difference data described above [14,15]. The percentages of nodes that showed consistent change between simulation results and expression difference data were calculated. The annotated expression difference data was obtained from the previous work [14,15] and it was listed in Table S4.

### 2.2. Cancer Gene Origin Analysis

The cancer driver genes were obtained from Cancer Gene Census (CGC) datasets [23] and Tokheim et al.’s work [24]. The latter provided cancer driver genes that were identified from 7916 distinct samples of 34 specific cancer types by eight prediction methods. Gene age information was obtained from Liebeskind et al.’s consensus gene age data [10]. These data divided human genes into eight classes: genes originating from the first cellular organism, common ancestor of eukaryota and archaea (euk_archaea), eukaryota, opisthokonta, eumetazoa, vertebrata, mammals and genes horizontally transferred from bacteria (euk+bac). The detailed information and references of these datasets are listed in the supplementary materials and Table S3.

The enrichment analyses were performed by comparing the percentage of cancer driver genes in each stage and the percentage of cancer genes in all genes, which were collected in the consensus gene age data. The significances of the deviations between the two percentages were tested by the hypergeometric test [25]. The test was corrected for multiple comparisons via a false discovery rate at the 0.05 level [26].

### 2.3. Cancer Prognosis Analysis

mRNA expression profiles of breast cancer, ovarian cancer, and glioblastoma multiforme patients and corresponding clinical information were downloaded from the Gene Expression Omnibus at the National Center of Biotechnology Information databases and The Cancer Genome Atlas (TCGA). The data processing and related references of these datasets are summarized in the supplementary materials and Table S3.

We chose gene signatures as biomarkers for cancer metastasis (or death) based on the enrichment pattern of cancer genes. The gene-age-based signatures were selected as follows. First, based on a training data set, Cox proportional hazards regression was applied to calculate the regression coefficient and *p*-value of every gene’s expression data with the metastasis or death risk of cancer patients. Genes with a *p*-value < 0.001 were selected as candidate genes. Next, we chose the genes originating from eukaryota, opisthokonta and eumetazoa from candidate genes as gene-age-based signatures. To evaluate the performance of the gene-age-based signatures, the most significant genes were selected as control signatures, and the number of these genes was the same as that of gene-age-based signatures.

With the gene-age-based signatures, we calculated the metastasis or death risk of each patient. The risk score of each patient was calculated with a strategy similar to the Gene expression Grade Index (GGI) [27], which was described as Equation (6):

(6) $$Risk\;Score=\sum g_{p}-\sum g_{q}$$

here *g_p_* is the expression value of gene with a positive regression coefficient (Cox proportional hazards regression), while *g_q_* is the expression value of genes with a negative regression coefficient. For the three breast cancer datasets, two ovarian cancer datasets and one glioblastoma multiforme data set, we classify the patients in each data set into high-risk group and low-risk group according to their risk score. At last, log rank test and survival analysis was performed by the Logrank matlab tool (http://www.mathworks.com/matlabcentral/fileexchange/22317-logrank).

## 3. Results

### 3.1. Simulation of Cancer Endogenous Molecular Networks Originating from Different Evolution Stages

The cancer endogenous molecular network theory was established to simulate the core regulation mechanisms underlying carcinogenesis. The main points of the theory were listed as follows.

First, the system controlling cancer phenotypes is constructed by the function modules that are related to cancer hallmarks. The modules could be represented by some key factors that play an important role in corresponding functions and the regulation relationship among these factors. The regulation relationship could be described by nonlinear stochastic equations and the network could be specified by the activities or concentrations of the factors, which resulted in a high dimensional stochastic dynamical system [11,12,13,14,15,16]. The system can represent the regulation mechanisms underlying carcinogenesis.

Second, the stable states (attractors) generated by the nonlinear stochastic dynamical system, which were represented by the sets of activities/concentrations of all the factors in the network, were assumed to be the states of real biological system that would stay in [11,12,13,14,15,16]. Cancer was also being regarded as an intrinsic state of the network. One can expect that the patterns of the nodes’ activities/concentrations in attractors generated by a reasonable network should be consistent with real cell status and this comparison was used to evaluate the accuracy of cancer endogenous molecular networks [14,15,16].

The cancer endogenous molecular network theory has been applied in four human cancers, including hepatocellular carcinoma [14], gastric cancer [15], prostate cancer [16] and leukemia [17] (In this work, we only used the networks of the three solid tumors, without leukemia). Due to the complexity and heterogeneity of cancer, it is nearly impossible to reproduce the actual network existing in an organism. So, in the above studies, simplified networks were constructed by functional modules corresponding to cancers’ hallmarks. The modules were composed by some nodes (proteins and protein complexes) which are key factors for the corresponding function. It has been proven that these simplified networks could reflect the mechanisms underlying carcinogenesis to a certain extent [14,15,16].

The cancer endogenous network is the product of evolution [12,13]. If genes originating in certain evolution stages are involved in carcinogenesis, one can expect that the functional parts originated in the corresponding stage are existed in the network. By investigating the subnetworks originating from certain evolution stages, we could trace the evolutionary origins of cancer driver genes in a systematic context. Considering that cancers have been observed in many different branches of multicellular organisms [28], it is of natural interest to investigate the behaviors of subnetwork originating from multicellular life. The multicellular subnetwork was constructed by the method as described in Section 2.1.1 and is shown in Figure S1. Compared with the complete networks, this subnetwork lacks nodes related to the immune response, such as cytokines (IFN-γ and TNF-α), interleukins (IL1, IL6, IL8 and IL10), and the related signal transducer (STAT3) (Table S2). The attractors of multicellular subnetwork were calculated as described in Section 2.1.2. The complete cancer networks constructed in the previous studies could generate four types of attractors which could characterize cell cycle arrest (be regarded as the normal-like status), stress response, proliferation (be regarded as the cancer-like status) and apoptosis [14,15,16]. Through comparing the relative activities/concentrations of each node in the multicellular subnetwork and the corresponding node in the complete networks, we found that the four attractors generated by the multicellular subnetwork were obviously in accordance with the four types of attractors generated by the complete cancer networks (Table S2). So, we thought the four attractors of our multicellular subnetwork could also characterize cell cycle arrest, stress response, proliferation and apoptosis, respectively. Given that the major topic of this study is carcinogenesis mechanism, our subsequent analysis was focused on the cancer-like and normal-like attractors.

To evaluate the accuracy of our result, the attractors generated by the subnetwork were examined by cancer gene expression datasets. This method has been used to investigate the accuracy of the complete cancer endogenous networks’ simulation [15,16]. The accuracy of the simulation result was calculated as we described in Section 2.1.3; we found it ranged from 63.0% to 85.3% in different datasets (Table S4), which was comparable to the simulation accuracy of complete networks (61.9% to 76.2% [15,16]). The details of the results were presented in Table S4. The accuracy of the simulation was also tested from the viewpoint of cancer driver gene types. According to the annotations of CGC, we found fourteen nodes composed of cancer driver genes. Among these nodes, nine contain oncogenes and show higher activation/concentration in the proliferation attractor. In the other five nodes, all contain tumor suppression genes, and four show lower activation/concentration in the proliferation attractor (Table S4). These results suggested that the simulation of multicellular subnetwork could reveal the mechanisms underlying carcinogenesis to a certain extent.

**Figure 1:**
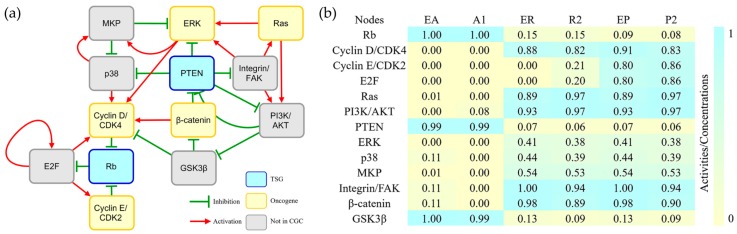
Eukaryotic subnetwork and its attractors (**a**) The network contains 13 nodes and 27 interactions. The colors of nodes indicate different cancer gene types: yellow for oncogene, blue for tumour suppressor genes (TSG), and grey for genes not collected in the Cancer Gene Census (CGC). The red arrows and green T-heads indicate activation and inhibition interactions, respectively; (**b**) The comparison of attractors of eukaryotic endogenous subnetwork and gastric cancer endogenous network. The numbers and color gradation are the normalized relative activities/concentrations that range from 0 (yellow) to 1 (blue) for minimal to maximal activity. A1, R2 and P2 are attractors of the gastric cancer endogenous network [15] that reflect cell cycle arrest, stress response, and proliferation, respectively. EA, ER and EP are attractors of the eukaryotic endogenous subnetwork, which are similar to A1, R2, and P2, respectively.

The above results are consistent with the current opinion that genes originating from multicellular organisms are an important source of cancer driver genes [4,5,6,7,8]. However, it remains unknown whether the cancer driver genes have an earlier origination history. From the multicellular subnetwork, we can find that its oldest part consists of 13 nodes originating from eukaryota (Figure 1a). The nodes of the subnetwork can be divided into different function modules. By investigating the KEGG pathways where these nodes are involved, we found that four nodes directly control cell cycle, and the rest participate in pathways related to cell cycle regulation (Table S5). The network simulation showed that three attractors of the eukaryotic subnetwork were similar to the attractors that reflect cell cycle arrest, stress response and proliferation (Figure 1b and Table S2). The accuracy of the simulation ranged from 69.2% to 92.3% calculated by the same cancer gene expression data mentioned above [15,16] (Table S4). These results suggested that the eukaryotic subnetwork could characterize the mechanism for the unlimited proliferation of cancer cells. In a control study, we tried to build endogenous networks without the eukaryotic part. Interestingly, all the resulting subnetworks failed to produce attractors consistent with the actual cell status (data not shown). Taken together, it is concluded that the oldest eukaryotic endogenous subnetwork recapitulates the basal hallmark of cancer, i.e., the unlimited proliferation.

It should be noted that the cancer endogenous networks which we construct in this study are vastly simplified symbols of mechanisms controlling cancer phenotypes. These networks only represent the general feature of cancer, and they are not suitable for analyzing the detailed mechanism of cancer, such as the intra-tumor heterogeneity.

### 3.2. Evolutionary Origins of Cancer Driver Genes

The endogenous network analysis suggested that genes originating from eukaryota may also be an important source of cancer drivers. However, the network only contains dozens of genes. To confirm this hypothesis, we need to investigate in a wider range. We resorted to cancer driver gene datasets, such as the CGC, which contains 581 manually selected cancer driver genes [23]. Besides, eight other datasets documenting predicted cancer driver genes were also used [24]. We investigated the distribution of cancer driver genes in evolutionary stages by enriching the genes with the consensus gene age data. In CGC, the significant enrichment was found in genes originating from eukaryota, opisthokonta and eumetazoa stages (Table 1 and Table S6). The enrichment in eukaryota and eumetazoa genes was also observed in six and five prediction datasets, respectively. Besides, opisthokonta was also found to enrich cancer driver genes in two prediction datasets (Table 1 and Table S6).

Previous studies have found the enrichment of the cancer-related genes in the genes originating from multicellular life. Using phylostratigraphy, Domazet-Lošo and Tautz tracked the origin of genes from three cancer gene datasets [6]. They reported two emergence peaks of cancer genes, one at the stage of the origin of cellular organisms which were related to the caretakers and showed indirect carcinogenesis, while the other at the stage of the origin of metazoan which were related to the gatekeepers and showed direct carcinogenesis [29,30]. Chen et al. compared the birth rate of CGC-annotated cancer driver genes originating from certain evolutionary stages and the birth rate of the randomly picked genes originating from the same evolutionary stages [7]. They detected a significantly higher birth rate of cancer driver genes in the transition stage of unicellular to multicellular organisms and in the early stage of metazoan. The enrichment of cancer driver genes in genes originating from opisthokonta and eumetazoa stages observed in this study is essentially the same with the results of the previous studies, while the enrichment in genes originating from eukaryota reveals another source of cancer driver genes. This observation was supported by the investigation focusing on the cancer genes used in the Domazet-Lošo and Tautz study (Table S7). Moreover, we investigated the distribution patterns of the caretakers and the gatekeepers. It was found that the enrichment pattern of the gatekeepers is the same with that of cancer driver genes, while the caretakers are enriched in the three earliest phylogenetic stages (Table S7). Thus, it seems that caretakers have an earlier origin than gatekeepers, in good agreement with the prior observation [6].

Then we investigated whether genes originating from eukaryota are involved in carcinogenesis in a different way from genes originating within multicellular organisms. The latter was thought to bring the collective fitness for the whole organism by suppressing the fitness of single cells, once this suppression was wakened, the cell may return to the unicellular state and become malignant [7,31]. Chen et al. have proven that loss-of-function of multicellularity-related genes could promote cancer metastasis in a xenograft tumour module [7]. We analyzed the functions of cancer driver genes annotated by CGC using DAVID [32] and found that the genes originating from opisthokonta and eumetazoa stages are related to the differentiation and development processes which were important to the maintenance of multicellularity (Table S8). Some of these genes code caspases, growth factors, and transcription factors, which participate in the multicellular subnetwork. However, the functions of genes originating from eukaryota are enriched in terms related to chromosome organization and cell cycle (Table S8). This result suggests that compared with genes originating with multicellular organism, genes originating from eukaryota play a different role in driving cancers through causing and keeping the activation of cell cycle.

### 3.3. Implications for Cancer Prognosis

Since cancer driver genes are of high value in cancer prognosis prediction [33], the present finding has direct medical implications. Cancer prognosis is critical for cancer treatment. Many works have attempted to predict the prognosis of cancer patients through statistical methods, which aimed to select genes that are related to cancer’s death or metastasis as gene signatures [34]. However, these signatures performed poorly in independent data sets [35]. This phenomenon may be caused by the high heterogeneity of cancer, which leads to many passenger genes being selected as gene signatures [33]. We speculate that the gene signatures selected by evolutionary considerations may remove passenger genes and thus improve the robustness of the prognosis models.

**Figure 2:**
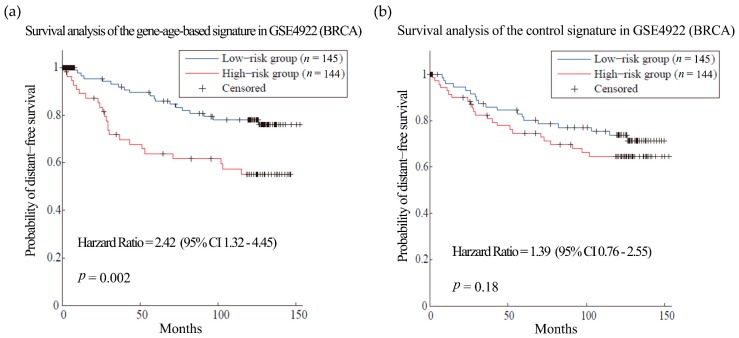
Survival analysis of the breast cancer (BRCA) dataset GSE4922. (**a**) and (**b**) are the predictions of the BRCA prognosis model using gene-age-based signatures and control signatures in independent test sets GSE4922 separately.

**Figure 3:**
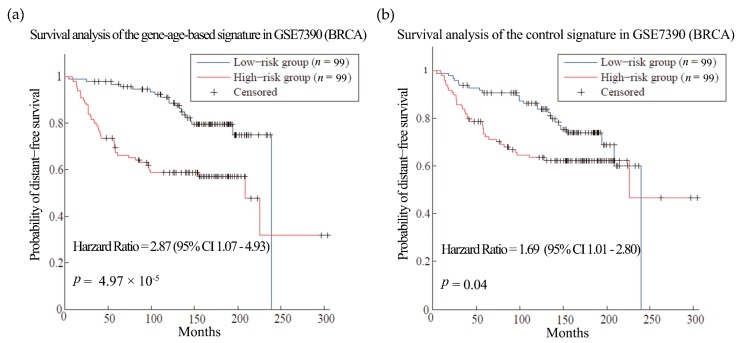
Survival analysis of the breast cancer dataset GSE7390. (**a**) and (**b**) are the predictions of the BRCA prognosis model using gene-age-based signatures and control signatures in independent test sets GSE7390 separately.

First, we tested our hypothesis in breast cancer (BRCA). BRCA is the most common female cancer worldwide and metastasis is the main cause of death among the patients [36]. Gene expression and clinical data of 286 patients from dataset GSE2034 [34] were used as training set to select the gene signatures of breast cancer metastasis. As a result, 135 genes were obtained as gene-age-based signatures (Table S9). We built prognosis models with the gene-age-based and control signatures separately and compared their performances in different datasets. In the training sets, the survival analysis showed that the gene-age-based signature is comparable with the models using control signature (Figure S2). Then we do the test in two other independent BRCA datasets. The gene-age-based signatures could predict the metastasis more accurately than the control signatures in both datasets: the hazard ratios (HR) of the gene-age-based signatures were 2.42 (*p*-value = 0.002) in dataset GSE4922 [37] and 2.87 (*p*-value = 4.79 × 10^−05^) in dataset GSE7390 [38], while the HR of the control signatures were 1.39 (*p*-value = 0.18) and 1.69 (*p*-value = 0.04) in the same datasets (Figure 2 and Figure 3).

**Figure 4:**
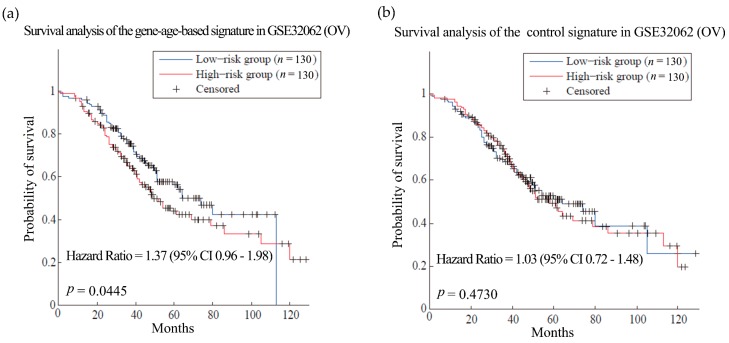
Survival analysis of the ovarian cancer dataset. (**a**) and (**b**) are the predictions of the ovarian cancer (OV) prognosis model using gene-age-based signatures and control signatures in independent test sets GSE32062 separately.

**Figure 5:**
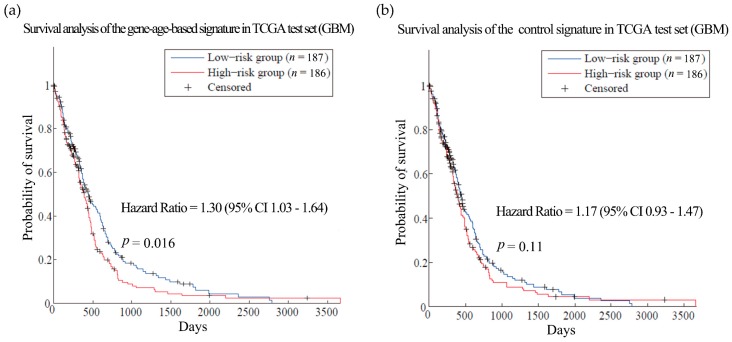
Survival analysis of the glioblastoma multiforme dataset. (**a**) and (**b**) are the predictions of the glioblastoma multiforme (GBM) prognosis model using gene-age-based signatures and control signatures in independent test sets in the GBM TCGA dataset separately. TCGA: The Cancer Genome Atlas.

Then the hypothesis was tested in ovarian cancer (OV) and glioblastoma multiforme (GBM). Patients with these cancers often have very short survival time [39,40]. Here, the training sets for the two kinds of cancer were composed of expression and clinical data from 567 OV patients and 200 GBM patients from TCGA, respectively [41,42]. We obtained gene-age-based signatures which include 61 and 58 genes separately (Table S9). Compared with the control signatures, the models established by the gene-age-based signatures made almost the same reliable predictions in the training sets (Figure S2). In the independent test set, the results of the gene-age-based signatures were HR = 1.37 (*p*-value = 0.0445) for OV (Figure 4, dataset GSE32062 [43]) and HR = 1.30 (*p*-value = 0.016) for GBM (Figure 5, TCGA test set [42]), which are more accurate than the results of the control signatures.

All of these results showed that the gene signatures selected by using evolution information exhibited better performance in discriminating the metastasis (or death) risks of cancer patients in independent data sets. This may be owed to the evolutionary filtering of passenger genes, and this filtering could improve the robustness of cancer prognosis prediction models. Though the module we used in this study is very basic, our result showed the practical utility of evolution information; more sophisticated models could be built based on our work. On the other hand, the confirmation of our hypothesis also supports that genes originating from eukaryota, opisthokonta and eumetazoa are important sources of cancer driver genes.

## 4. Conclusions

In summary, tracing the origins of cancer driver genes in the endogenous molecular networks provides us with a deeper understanding of the link between cancer and macroevolution. The simulation of endogenous subnetworks originating from multicellular life and eukaryota provides a systematically characterization for the carcinogenesis of genes originating from different evolutionary stages. In addition, analysis of different cancer driver gene datasets showed robust enrichments of cancer drivers in genes originating from eukaryota, opisthokonta and eumetazoa. These findings shed new light on the evolutionary origins of cancer driver genes. This information can be used to filter passenger genes, and thus can improve the robustness of cancer prognosis prediction models.

## Figures and Tables

**Table 1:** Enrichment pattern of cancer driver genes.

Cancer Driver Gene Datasets	1	2	3	4	5	6	7	8
20/20+ (197)				***		*		
ActiveDriver (412)		*		***	**	**		
CGC (581)				**	**	***		
MuSiC (1952)					**	***	***	
MutsigCV (156)				***				
OncodriveCLUST (570)							***	*
OncodriveFM (2560)	***	*	***	***				
OncodriveFML (669)				***		**		
TUSON (243)				***		***		

The information of cancer driver genes was obtained from the Cancer Gene Census (CGC) [23] and Tokheim et al.’s work [24]. The datasets and the numbers of genes with age data contained in each dataset were listed in the first column (on the left). The gene age information was obtained from the consensus gene age data [10]. The columns entitled 1 to 8 represent the following evolution stages: cellular organisms, euk_archaea, euk+bac, eukaryota, opisthokonta, eumetazoa, vertebrata and mammalian. Statistical significance of the enrichment was tested by the hypergeometric test corrected for multiple comparison by a false discovery rate at 0.05 level (* *p* < 0.05; ** *p* < 0.01; *** *p* < 0.001).

## Supplementary Materials

The following are available online at www.mdpi.com/2073-4425/8/7/182/s1. Figure S1: The multicellular subnetwork, Figure S2: Survival analysis on training sets, Table S1: Regulatory relationships in multicellular and eukaryotic subnetwork, Table S2: Comparison of attractors of multicellular subnetwork, attractors of eukaryotic subnetwork and attractors of complete cancer networks, age of nodes in networks, Table S3: Sources of the datasets used in this study, Table S4: Comparison of the simulation results of multicellular and eukaryotic subnetworks with the related experimental results, Table S5: The eukaryotic-subnetwork-nodes related Kyoto encyclopedia of genes and genomes (KEGG) pathways, Table S6: Gene age information and enrichment pattern of cancer driver genes from different datasets, Table S7: Gene age information and enrichment pattern of cancer genes used in the Domazet-Lošo and Tautz study [6], Table S8: Gene ontology (GO) enrichment of cancer driver genes originating from different evolutionary stages, Table S9: Gene signatures in cancer prognosis analysis.
